# Case report: Emergency management of spontaneous rupture of the inflammatory myofibroblastic tumor of the urinary bladder

**DOI:** 10.3389/fonc.2022.948263

**Published:** 2022-11-15

**Authors:** Zhenfu Sun, Lei Qi, Zhifan Guo, Wei Yuan, Yuancheng Du, Haitao Gao, Xuekai Hong, Yunjiang Zang

**Affiliations:** ^1^ Department of Urology, Weifang Medical College (University), Weifang, Shandong, China; ^2^ Department of Urology, Weifang People’s Hospital, Weifang, Shandong, China; ^3^ Department of Urology, Affiliated Hospital of Weifang Medical College, Weifang, Shandong, China

**Keywords:** IMTUB, spontaneous rupture, pathology, partial cystectomy, bladder tumor

## Abstract

Acute abdomen caused by spontaneous rupture of the inflammatory myofibroblastic tumor of the urinary bladder (IMTUB) is a rare clinical emergency of the urinary system.It is difficult to distinguish it from spontaneous rupture of bladder caused by other causes before surgery. An emergency case of spontaneous rupture of IMTUB was reported. A 57-year-old middle-aged woman was admitted to the hospital because of “acute lower abdominal pain for 5 hours”. No history of smoking and gross hematuria. The physical examination revealed visible abdominal tenderness as well as signs of shock. A pelvic CT scan shows a space-occupying lesion above the bladder with massive accumulation of blood. When the nature of the tumor could not be determined, emergency laparotomy and partial cystectomy were performed, and postoperative pathology confirmed cystitis myofibroblastic tumor. No local recurrence or distant metastasis of the tumor was observed during the regular follow-up period of 6 months. IMTUB should focus on prevention and treatment, with a combination of preoperative examination and postoperative pathology, and finally implement highly individualized treatment.

## Introduction

Inflammatory myofibroblastic tumor (IMT) is a rare clinical benign mesenchymal tumor with uncertain malignant potential that can occur in the lung, gastrointestinal tract, liver, abdominal wall, or retroperitoneal soft tissue, among other places (1), and less common in the urinary system (2). IMT is more common in adolescents and young adults and is more likely to affect men than women, children and the elderly are rarely affected (3, 4).

The clinical presentation of inflammatory myofibroblastic tumor of the urinary bladder (IMTUB) is characterized by episodic painless hematuria (2). The pathogenic mechanism of IMTUB is unknown, but some researchers believe that it is linked to infection (bacteria and virus), autoimmunity and genetic factors (5). In clinical practice, acute abdomen caused by spontaneous rupture of IMTUB is rare.

We present a case of acute abdomen caused by spontaneous rupture of IMTUB. It provides a reference for the emergency diagnosis and treatment of spontaneous rupture and bleeding of IMTUB by reviewing its diagnosis and treatment process, in combination with relevant literature reports.

## Case description

**Figure 1:**
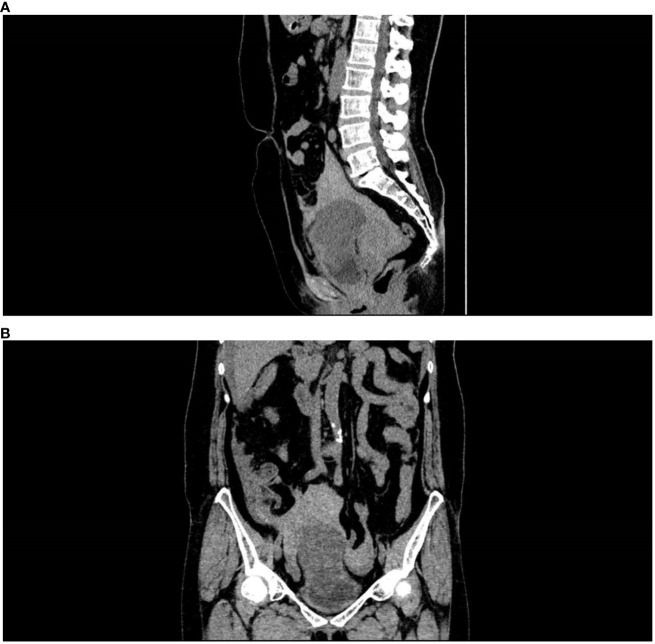
Enhanced CT scan shows pelvic hemorrhage. **(A)** Side view, **(B)** Front view.

The patient, a 57-year-old female farmer, came to the clinic on August 14, 2021. She developed severe pain in the lower abdomen with no obvious cause 5 hours ago, accompanied by nausea and vomiting. The outpatient was admitted to the hospital because of “abdominal pain, the cause of which is being investigated?” On admission, the blood pressure was 88/56 mmHg, and signs of shock were visible. Physical examination revealed a distended abdomen with obvious tenderness but no rebound tenderness. A pelvic CT scan showed that the bladder can be filled, there is a 7.4cm-diameter spherical kind of oval low-density shadow above the bladder and a space-occupying lesion above the bladder, as well as large number of pelvic lesions with massive accumulation of blood about 1000ml. (**Figures 1A, B**) The initial diagnosis was bladder mass and pelvic hemorrhage based on the patient’s symptoms and auxiliary examinations, and the specific cause is unknown.

During the preoperative examination process, the patient’s blood pressure and blood oxygen saturation decreased gradually, while the shock gradually increased. Following an assessment of the patient’s basic condition, an emergency laparotomy and partial cystectomy were performed. During the procedure, a convex tumor of covering serosa was discovered on the top wall of the bladder. On the surface of the mass, a 3-cm-long fissure with persistent bleeding and tumor tissue extravasation can be seen. There were numerous blood clots in the pelvis, and the mass and bowel were heavily adhered. After we explained to the patient’s family what the doctor saw during the operation, the family agreed to let us perform a partial cystectomy on the patient. There was complete resection of a well-defined tumor two centimeters along the tumor margin, with normal bladder tissue. The intraoperative removal of blood clots was approximate 1300ml, and fresh bleeding was approximate 200ml. Following the operation, the patient recovered well. Every three months, a pelvic CT scan and a cystoscopy were performed. During the 6-month follow-up, no local recurrences or distant metastasis were discovered.

**Figure 2:**
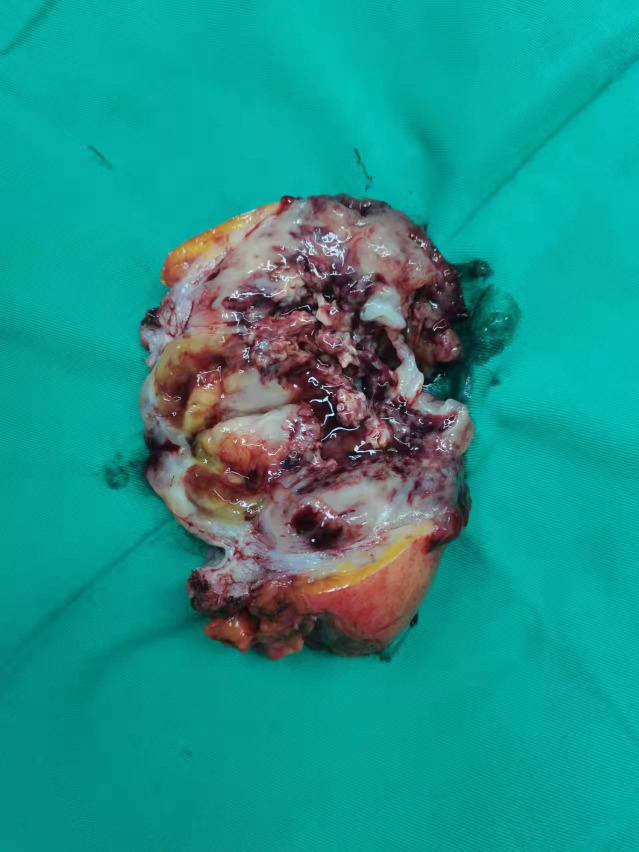
Macroscopic view of pathological specimens.

**Figure 3:**
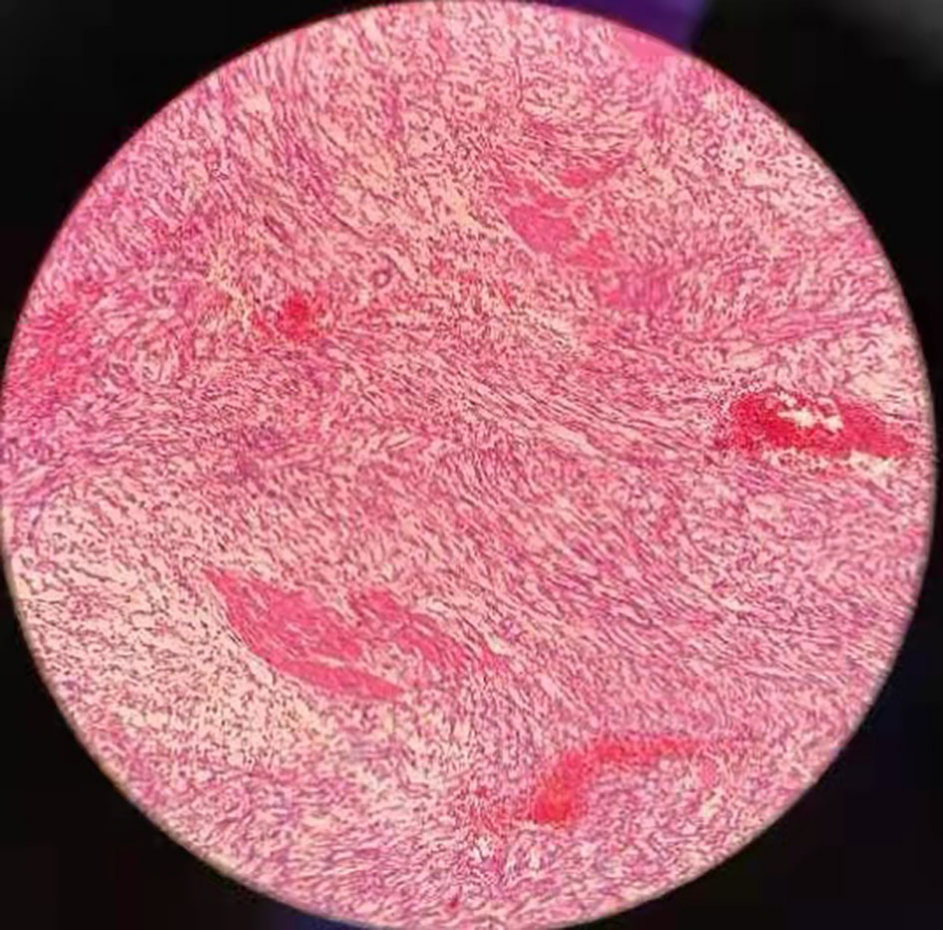
Final pathology results showed extensive fibrocytic and inflammatory cell infiltration.

Gross pathology showed a 12*6.5*4 cm tumor that was gray-white, gray-red and translucent. (**Figure 2**) Microscopic examination showed spindle cell tumor with mucinous background, chronic inflammatory cell infiltration, and erythrocyte extravasation, with unclear local boundaries and invasion of surrounding muscle tissue. Immunohistochemical findings: CK broad (+), Vimentin (+), ALK (+), SMA (partially weak +), Desmin (partially +), S-100 (-), Ki-67 index (5%)(**Figure 3**). The MDM2 gene was not amplified in the FISH test, so the result was negative. Combined with the results of immunohistochemistry and FISH, it was consistent with inflammatory myofibroblastic tumor. When the patient’s diagnosis and treatment process were combined, as well as the postoperative pathological results, the patient was finally diagnosed as having acute abdomen caused by spontaneous rupture and hemorrhage of IMTUB, and partial cystectomy has a good effect on the treatment of this disease.

## Discussion

The onset of inflammatory myofibroblastic tumor of the urinary bladder (IMTUB) is relatively slow, and patients frequently present with intermittent or persistent painless gross hematuria as the first symptom (6), with lower urinary tract irritation symptoms occurring in some cases (1). It is extremely uncommon to arrive at the hospital with severe abdominal pain as the first symptom. Clinically, the posterior wall of the bladder (33.3%) was the most common site of IMTUB, followed by the top wall (22.2%), right wall (19.4%), left wall (13.9%), anterior wall (11.1%) and Triangle (0%) (7).

IMTUB is typically seen as a smooth-walled, wide-basal lesion rather than a papillary lesion. Lamovec accurately described the IMTUB histological pattern. The tumor’s luminal surface was edematous and focal hemorrhagic fibroblast tissue, with a large number of capillaries and sparsely cellular fibromatous tissue, accompanied by inflammatory cells, plasma cells, and/or lymphocytic infiltration (8). The anatomical location and histology differences of IMTUB provide a theoretical basis for rapid intraoperative diagnosis and identification. The histological morphology of IMTUB, on the other hand, makes early detection of cystoscopy and microscopic biopsy difficult.

Pathological findings are still the gold standard for determining IMTUB. Vimentin, ALK, and SMA are mostly diffusely positive in IMTUB pathology, but not S100, CD117, CD34, STAT6, and so on (1, 9). ALK is rarely expressed in bladder spindle cell tumors other than IMT, according to a number of studies conducted in China and the United States (10, 11). Ki-67, a nuclear protein associated with ribosomal RNA transcription, is a cell proliferation marker that is highly expressed in cancer cells.

Ki-67 positivity is an important factor in predicting benign and malignant tumors, recurrence, and progression, and higher Ki-67 expression often indicates a higher malignant tendency of tumors (1) and is associated with higher recurrence rate, higher invasiveness, and shorter cancer-specific survival of tumors(CSS). According to some researchers (7), preoperative histopathology can distinguish IMTUB from other benign and malignant bladder tumors, and postoperative pathological results can evaluate patients’ prognoses and provide a reliable basis for the next treatment and postoperative follow-up.

In this case, the patient developed hypovolemic shock as a result of spontaneous rupture and bleeding of the IMTUB and required an emergency partial cystectomy. The patient received good surgical benefits, according to postoperative pathology and follow-up results. Currently, surgery will provide the greatest benefit for this condition, and the clinical treatment of IMTUB is highly individualized, and the common surgical methods include transurethral resection of bladder tumor (TURBT), partial cystectomy and radical cystectomy. Some researchers have performed intravesical instillation of anti-inflammatory drugs, but no clinical significance has been established (12). The WHO classifies IMTUB as a tumor with intermediate biological potential (13), with a metastasis and recurrence rate as low as 4%, but its potential for metastasis and recurrence remains a source of contention (14). Only one case of IMT tumor cell infiltration into the fat surrounding the bladder was observed in cystectomy samples, with tumor tissue extending locally to the adjacent colon without distant metastasis (14). At the moment, the most common treatment for IMTUB is electric resection or partial cystectomy, with only a few cases of radical cystectomy reported (15). Teoh (7) and others systematically reviewed the follow-up of 120 patients with IMTUB after various treatments. Among them, 60.8% received initial resection of bladder tumor (TURBT), but 24.7% had local recurrence and required further treatment. Treatment, which included partial cystectomy (17.8%), second TURBT (5.5%), and radical cystectomy (1.4%). In 29.2% of patients, a partial cystectomy was performed, 9.2% had a radical cystectomy, and 0.8% had a cystoscopic biopsy. 60% of IMTUB postoperative pathological findings revealed invasion of the bladder’s muscularis propria. During the follow-up period, no tumor recurrence or distant metastasis was discovered. The high recurrence rate following TURBT does not distinguish between true tumor recurrence and postoperative tumor residual.

This case demonstrates that, in addition to advocating for individualized treatment, IMTUB treatment is more important to early diagnosis and intervention. Because of the patient’s lack of awareness of physical examination, the patient’s final illness was delayed, and the acute abdomen caused by spontaneous rupture and bleeding of IMTUB posed a serious threat to the patient’s life and made diagnosis and treatment difficult. We recommend that patients with abdominal pain, gross hematuria, abdominal mass, or other unpleasant symptoms seek medical attention and treatment as soon as possible to avoid delaying treatment. The ultimate goal is to accomplish the goal of early detection and treatment.

Due to the difficulty of biopsy under the microscope with IMTUB, TURBT can be used for pathological examination to determine the nature of the tumor and choose a surgical plan. When the early examination found that the tumor was small, single, and the first electroresection pathology shows no muscle layer invasion, the bladder tumor electroresection (TURBT) is a viable option. However, due to its relatively high recurrence rate, regular follow-up of cystoscopy and pelvic CT are required every 3 months after the operation. Partial cystectomy is recommended when the tumor is large and the pathology shows that the muscle layer has infiltrated, but the tumor can still be completely removed. Radical cystectomy is an option when the tumor is multiple in the bladder or has occupied most of the bladder and cannot be completely removed, However, the quality of life and psychological problems of patients after total cystectomy must be closely monitored. As a result, partial cystectomy has become the preferred surgical method for treating IMTUB.

In terms of clinical symptoms and signs, spontaneous IMTUB rupture is difficult to distinguish from spontaneous bladder rupture. It can be distinguished from clinical symptoms and imaging examination. The first signs of spontaneous bladder rupture include severe abdominal pain, massive hematuria or microscopic hematuria, as well as obvious local tenderness, positive rebound tenderness or no obvious signs. The CT plain scan shows poor bladder filling, and significant contrast agent leakage can seen in the pelvis. The main cause of its occurrence is the pathological changes of the bladder itself, such as tumors and diverticulum (16). At the early stages of IMTUB, there was only a small amount of microscopic hematuria and slight lower abdominal pain. Only when the tumor ruptured, there was severe abdominal pain. The CT examination shows that the bladder is well filled, and generally there is no leakage of contrast medium. When it breaks, tumor tissue can overflow. At present, spontaneous bladder rupture can only be identified by intraoperative exploration and postoperative pathology, so it is expected that there will be more accurate inspection and inspection methods in the future to improve the detection rate of IMTUB.

## Summary

IMTUB is a rare disease, and spontaneous rupture requires a combination of preoperative examination, intraoperative findings, and postoperative pathology, as well as multiple differential diagnoses. It should be considered as one of the causes of spontaneous bladder rupture. Before surgery, the spontaneous rupture of IMTUB must be distinguished from other diseases by combining the medical history and auxiliary examination. Among many treatment options, partial cystectomy stands out for its low recurrence rate, quick recovery, and high quality of life. The final surgical method should be determined by imaging analysis, preoperative histopathology, and intraoperative conditions. The pathological results of TURBT are especially important for the selection of surgical methods in non-emergency patients with IMTUB. Early examination, regular review, and individualized treatment are recommended for patients with unexplained abdominal pain and hematuria. To improve the detection rate of IMTUB, future research should focus on the detection of blood and urine markers, as well as more targeted imaging examinations.

## Data availability statement

The original contributions presented in the study are included in the article/supplementary material. Further inquiries can be directed to the corresponding author.

## Ethics statement

Ethical review and approval was not required for the study on human participants in accordance with the local legislation and institutional requirements. The patients/participants provided their written informed consent to participate in this study. Written informed consent was obtained from the individual(s) for the publication of any potentially identifiable images or data included in this article.

## Author contributions

ZS, LQ and YZ have made substantial contributions conceptions and design and have been involved in drafting the paper. ZG, HG and XH have performed data acquisition. ZS, YD, WY revised the manuscript. All authors contributed to the article and approved the submitted version.

## Acknowledgments

The authors thank the family for participating in the study.

## Conflict of interest

The authors declare that the research was conducted in the absence of any commercial or financial relationships that could be construed as a potential conflict of interest.

## Publisher’s note

All claims expressed in this article are solely those of the authors and do not necessarily represent those of their affiliated organizations, or those of the publisher, the editors and the reviewers. Any product that may be evaluated in this article, or claim that may be made by its manufacturer, is not guaranteed or endorsed by the publisher.
